# Insights on Three Dimensional Organoid Studies for Stem Cell Therapy in Regenerative Medicine

**DOI:** 10.1007/s12015-023-10655-6

**Published:** 2023-12-14

**Authors:** Precious Earldom Mulaudzi, Heidi Abrahamse, Anine Crous

**Affiliations:** Laser Research Centre, Faculty of Health Sciences, University of Johannesburg, P.O. Box 17011, Doornfontein, 2028 South Africa

**Keywords:** Regenerative Medicine, Organoids, Stem Cell Therapy, Three-dimensional Cell Culture

## Abstract

Regenerative medicine has developed as a promising discipline that utilizes stem cells to address limitations in traditional therapies, using innovative techniques to restore and repair damaged organs and tissues. One such technique is the generation of three-dimensional (3D) organoids in stem cell therapy. Organoids are 3D constructs that resemble specific organs' structural and functional characteristics and are generated from stem cells or tissue-specific progenitor cells. The use of 3D organoids is advantageous in comparison to traditional two-dimensional (2D) cell culture by bridging the gap between in vivo and in vitro research. This review aims to provide an overview of the advancements made towards regenerative medicine using stem cells to generate organoids, explore the techniques used in generating 3D organoids and their applications and finally elucidate the challenges and future directions in regenerative medicine using 3D organoids.

**Graphical Abstract:**

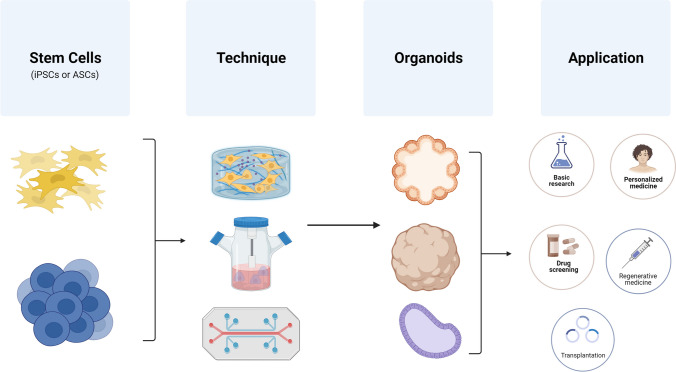

## Introduction

Regenerative medicine, representing a new and emerging area of research in the field of health science studies, focuses on the generation and development of specific functional biological substitutes for restoring, replacing, or improving tissues and organ function [1]. It encompasses three subcategories, namely, bioartificial organs, tissue engineering, and cell therapy. Tissue engineering focuses on the use of cells to regenerate biological tissue with the assistance of supporting structures and/or biomolecules [2, 3]. While cell therapy focuses on the use of cell culture to improve, maintain, and/or restore the functionality of tissues and organs [4].

Stem cell therapy has become a major concept associated with regenerative medicine [2]. Stem cells have been used in various sub-categories of regenerative medicine. Stem cells are undifferentiated cells able to differentiate into many types of different specialized cells and tissue [3] as well as self-renew through cell division [4]. The ability of stem cells to divide, differentiate and develop into different specialized cell types and their capacity for constant self-renewal make them ideal candidates for various kinds of stem cell based therapeutic applications [5]. Stem cells can be found in embryos and adult tissue [5]. Depending on whether they are primarily embryonic or present in post-embryo adult tissues the specific characteristics of stem cells with respect to the capacities for self-renewal, division and differentiation have been classified as totipotent, pluripotent, multipotent, or unipotent cells [6]. Different stem cells have previously been identified, namely: embryonic, induced pluripotent and adult stem cells, depending on where they have been isolated [5]. Embryonic stem cells (ESCs) are isolated from a blastocyte, induced pluripotent stem cells (iPSCs) isolated from programmed adult stem cells and adult stem cells (ASCs) isolated from mature tissue [7]. Pluripotent stem cells can under specific conditions differentiate into any cell type in the body and includes both embryonic stem cells (ESCs) and induced pluripotent cells (iPSCs) [8]. Adult stem cells are tissue-specific stem cells that can be isolated from mature adult tissue and possess the ability to self-renew and differentiate into specific cell types [10, 12, 14]. Organoids have previously been generated from both ASCs and PSCs [9, 10] (Fig. 2B).

To date researcher are working towards the development of 3D cancer stem cells to address challenges faced in culturing delicate differentiated cells and non-malignant stem cells in 2D [11]. The use of 3D culture in cancer studies allows for assessing the pathophysiology of cancer progression and resistance [12], replicating a patient's tumour in vitro to enable individualized treatment screening [13] as well as screening anti-cancer treatments in vitro [14]*.* In 2013 Kimlin and colleagues demonstrated that cancer stem cells in 3D are able to mimic the recurrence conditions and also show for a realistic treatment response [15]. The promise that exists now is that cells cultivated in three-dimensional aggregates have the capacity to increase resistance to different cancer treatments. Recent research by Narmi et al. in 2023, which discovered that using 3D culture is an efficient way to evaluate anticancer drugs, has supported this work [16]. Various priming techniques have been developed that aim to sensitize cells and get them ready for therapeutic treatment. The priming of normal and cancer stem cells differs significantly when comparing 2D and 3D culture. 3D preparations of cancer stem cells may be used to evaluate combination therapies or sensitization agents that target both bulk tumour cells and resistant stem cell populations. This provides a more representative platform for researching these methods [11, 17]. In 2023 Narmi and colleagues discovered that the use of melatonin in three-dimensional culture has significant promise for comprehending the anti-tumour activity exhibited by melatonin in reaction to certain angiogenesis elements [16].

Techniques have been developed for generating organ specific tissues in the form of organoids from specific stem cells which can be used for the regenerative treatment of diseased or damaged organs. These techniques have focused on the generation of tissue and organ specific organoids from various stem cell sources [7]. Organoids are small, self-organizing, three-dimensional (3D) tissue culture structures that have been generated from stem cells and can be propagated in vitro under various tissue culture procedures [8, 18]. Organoids as model tissue culture systems possess multiple growth and development attributes, such as self-organization and self-renewal, and capacities to perform functions similar to those of the tissues they mimic [19]. The science of regenerative medicine can be advanced by using organoids to research the mechanisms of development and regeneration through organ modelling [20]. The purpose of this review is to present the recent advances that have been made in the techniques and procedures for the induction, generation and development of organoids from various stem cell sources, as well as the advances in the therapeutic applications of organoids in organ regenerative medicine.

## Modern Regenerative Therapies Using Three-Dimensional Organoids

Previously, the basic cell and molecular biology, as well as the physiology, underlying the mechanisms responsible for the maintenance of the structure and functioning of the human nervous system were studied under in vitro conditions using 2D tissue and cell cultures. However, these 2D model systems based on in vitro cell and tissue culture procedures display organization features and physiological functions that are different from those occurring in vivo [21]. This is because they lack the 3D structural and functional organization that forms the basis for the cellular and physiological connections between various types of neural cells [22, 23]. Traditionally 2D monolayer cell cultures have been used in drug discovery and a wide range of clinical research [24]. Primarily cell cultures are generated on a flat glass or plastic surface [25]. In 2009 Dutta showed that growing cells on a 2D monolayer does not sufficiently demonstrate the natural in vivo micro-environment [26], this method has always been assumed to mimic in vivo cell growth [27]. Studies suggest that the use of 2D cell and tissue culture has been shown to have some limitations, for example cells maintained in a 2D environment may lose several cell-specific characteristics seen in living organisms, such as shape, polarity, differentiation, and metabolic profile [27–29]. The biggest disadvantage of using 2D cell culture is that the cell lines are unable to mimic the cellular structural and functional characteristics of functional tissues or organs, and the main reason for this is because they lack the required cell-to matrix and cell -to-cell interaction [18]. With current limitations using 2D cultures still requires the use of animal however, testing the use of animals has risen an ethical concern due to pain and discomfort experienced by the animals [24].

**Fig. 1:**
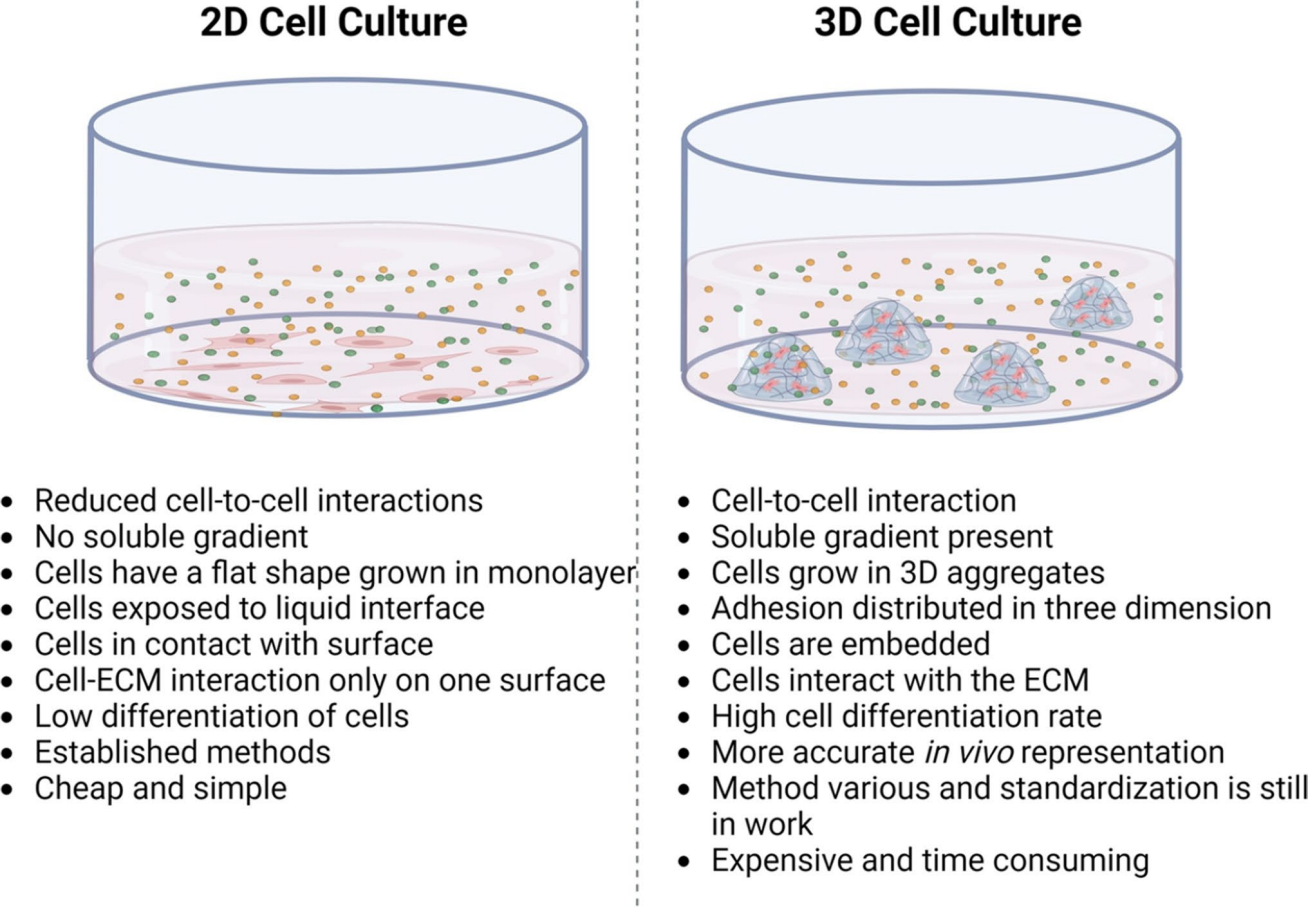
Comparison of 2D and 3D cell culture system

The use of and reliance on 2D cell cultures as model systems in pre-clinical research has led to a knowledge gap between pre-clinical research and clinical research, as the observations and findings of the former are subject to significant limitations and shortcomings which cannot be used to elucidate and make predictions at a clinical level [30, 31]. Consequently, pre-clinical research studies need to be based on procedures that involve the use of 3D cell and tissue cultures that are cultured and maintained under conditions that replicate the in vivo physiological environment [32, 33]. Over the years the development of new cell culture techniques that enable 3D cell growth have been developed to address these drawbacks [27]. Three-dimensional cell cultures can produce an artificial extracellular micro-environment (ECM) where cells develop and interact with their environment in three dimensions and enables the modelling of more complex organ-like structures as well as different cell types at the same time [18, 34–36]. The differences and similarities between the two cell culture methods have been reviewed in detail previously [24, 37, 38], Fig. 1 shows comparison of 2D and 3D cell culture systems.

The signalling, pathways involving paracrine and juxtacrine systems comprise an extensive network that intricately governs cellular behaviours and interactions, notably in the microenvironment of stem cell regulation [39, 40]. Paracrine signalling is the production of signalling molecules by one cell to target adjacent cells, altering stem cell activity such as proliferation and differentiation by use of growth factors and cytokines [41]. While direct cell-to-cell contact is used in juxtacrine signalling, which allows for precise control of stem cell characteristics like as migration and differentiation [42]. The paracrine signalling is limited in 2D culture because of lack of spatial organization, diffusion of paracrine factors is seen as restricted and this results in the limitation of cell-to cell contact [21, 43]. While in 3D culture the is increased cell to cell contact and increased spatial organization allowing for increased diffusion of paracrine factors as well as prompting cell-to-cell communication [44]. The juxtacrine signalling mechanism by which direct cell-to-cell contact and signalling is facilitated increases in 3D culture as there is an increase in the spatial organization and promotes ligand-receptor interaction allowing for a direct signalling pathway to exist between the cells [40, 43]. In 3D culture system there is an increase in the pathway involving the paracrine and juxtacrine signalling due to increase in cell-to-cell communication and reduced diffusion distance. The study of paracrine and juxtacrine signalling pathways in stem cell behaviour particularly in 3D cell culture is promising for advancing our understanding of cellular mechanisms and unlocking stem cell potential in regenerative medicine applications.

In 2015, Li and colleagues tried to establish a 3D system for the culturing of mesenchymal stem cells derived (MSCs) from the human umbilical cord within a real 3D microenvironment. The obtained results showed that cell-to-cell and cell-to-matrix connections are easily made in 3D cultures and MSC communication with the surrounding cells increased [45]. The findings of this work have further been confirmed by additional studies which have demonstrated that the use of 3D cell culture can bridge the experimental gap between in vitro cell culture and in vivo animal models [46]. Three-dimensional cultures are immortalized stem cells or cell lines which are arranged inside hydrogel matrices to resemble an in vivo cell environment [33]. In 2017, Simian and Bissell reported that the in vitro growth of 3D cellular structures could be classified as organoids. However, the precise definition of an organoid is still under review [19]. Organoids can be generated using various methods and from either PSCs or ASCs [46–48]. To date, many researchers have performed various experiments on the generation of organoids from either PSCs or ASCs. In 2022, Tang and colleagues reviewed the difference in organoids generated from PSCs and ASCs. They found that PSC-derived organoids are more suitable for researching early organogenesis because they form exclusively during embryonic development. However, ASC-derived organoids provide a better understanding of tissue and organ repair as well as studying disease [18]. Although both PSCs and ASCs could be used to generate organoids, the use of ASCs has been shown to be advantageous because they are obtained directly from human adult tissue with simpler procedures [49, 50].

## Role of Three-Dimensional Organoids in Stem Cells Therapy

Since the development of organoids, they have been shown to be advantageous in the application of stem cell therapy. Scientists have since used organoids to study the function of stem cells in tissue regeneration, maintenance, communication and disease modelling [51, 52]. Organoids provide a more accurate representation of the in vivo physiology of the organ system and offer a stable system for tissue regeneration [46, 53]. They mimic biological characteristics like spatial order, cell–cell communication, and physiological processes of the cells, thereby filling the gap in knowledge that exists between the natural physiological environment and the in vitro environment [47, 54]. Organoids are increasingly being used for modelling diseases because they contain a variety of cell types and are not constrained by interspecies differences [55]. Table 1 indicates a few diseases that have been modelled using stem cell derived organoids.

**Table 1:** Diseases model using 3D Organoids derived from various stem cells

Organoid	Disease Modeled	Method Used	Cell Source	Days Generated	Reference
Brain	Zika virus	Matrigel and Orbital sharker	Human embryo	65 + days	[56–58]
	Autism spectrum disorder	Matrigel and Orbital sharker	stem cells	45 days	
	Alzheimer’s	Matrigel	hiPSCs hiPSCs	60 days	
Pancreas	Cystic Fibrosis	Matrigel and microfluidics	Patient derived and hiPSCs	10 + days and 30 days	[59–62]
	Pancreas cancer	Matrix gel	Patient derived	-	
	Diabetes	Matrigel	hiPScs	90 + days	
Prostate	Prostate cancer	Matrigel	Patient derived	-	[63, 64]
Lung	SARS-COV-2 COVID 19	Matrigel	Patient derived	30 days	[65]
Heart	Cardiomyopathy	Matrigel	hiPSCs	21 days	[66, 67]
	Heart disease	Matrigel	hiPSCs	56 days	
Kidney	Polycystic kidney disease	Geltrex	hiPSCs	25 + day 35 + days	[68, 69]

## Organoids in History

**Fig. 2:**
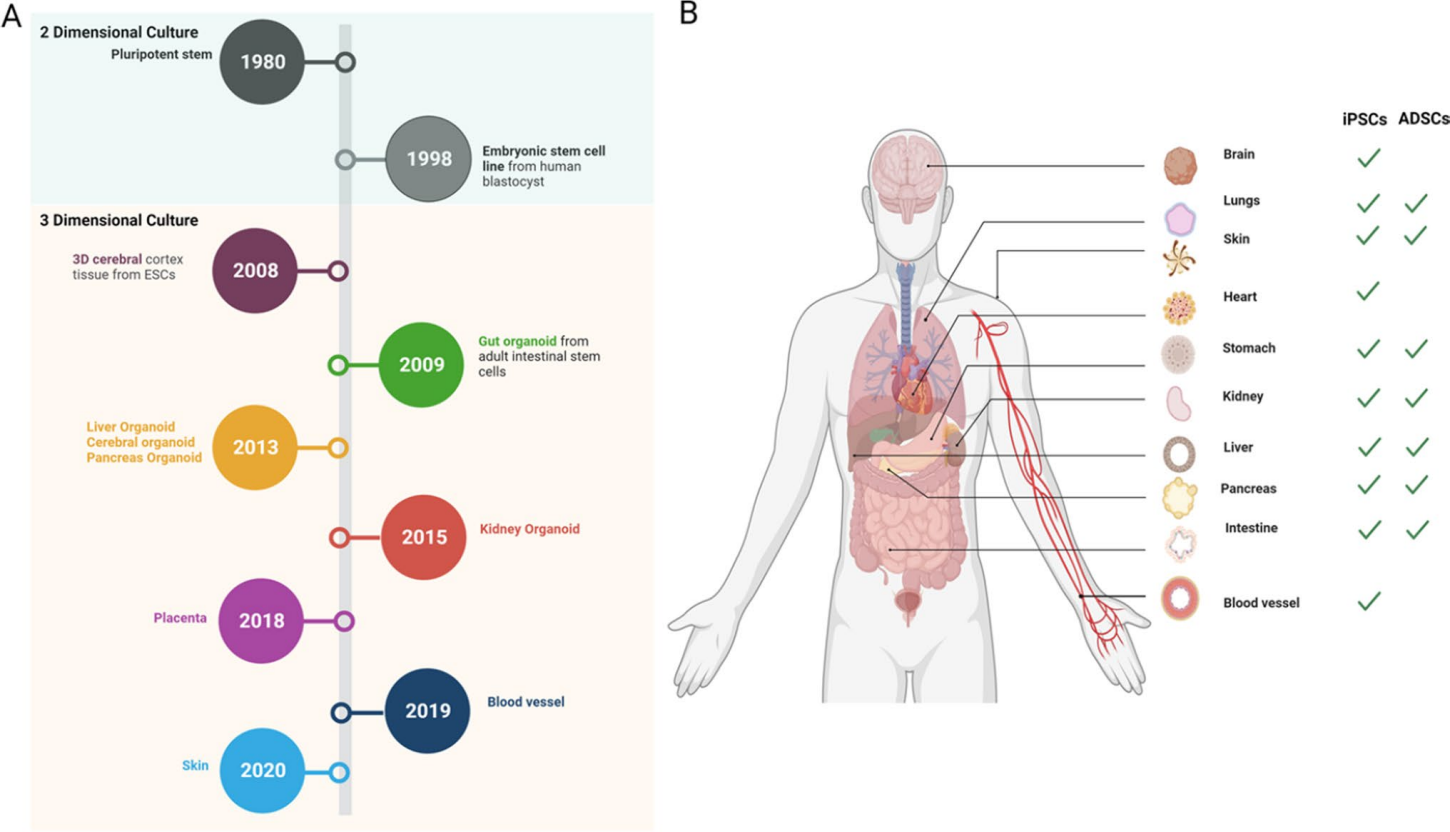
Organoid generation. **A** Depletes the histological timeline on the development of organoids. **B** Show the various organoids that have been generated to date and the stem cells used to generate these organoids

The field of stem cells has flourished greatly since the discovery of pluripotent stem cells from mouse embryos in 1980 [70]. In the 1990’s, researchers were successfully able to separate and culture embryonic stem cells from human blastocysts [71]. In 2008, stem cell research started shifting from 2 to 3D when Erika et al. generated 3D central cortex tissue from ESCs [72]. Building on the work by Erika and colleagues in 2009; Sato et al. developed the first generation of intestinal organoids [73]. Since the discovery of organoids in 2009, considerable work has shifted from 2D into 3D generating organoids (Fig. 2). Disease models are increasingly being developed using organoids. Novel therapy techniques based on the utilization of organoids have been developed [46, 74].

In 2009, Sato and colleagues reported how single Lgr5 + stem cells could be used to generate the first long-term 3D culture of intestinal organoids. Organoids were grown on a Matrigel supplemented with various growth factors until differentiation into intestinal cell types. The obtained results were used to set the groundwork for developing the technology that could be used to study various diseases in vitro [75]. Building on this work Spencer and colleagues went on to show that human induced PSCs and ESCs can be differentiated into functional 3D intestinal organoids in vitro using Matrigel matrix supplemented with growth factors [76].

In 2013, building on the foundations of the work started by Sato, Huch, and colleagues, Spencer and colleagues performed an in vitro expansion of Lgr5 + liver stem cells. In this mouse model, the damaged liver of a mouse was extracted and cultured into 3D organoids on Matrigel. The obtained results from this study showed that the cells could differentiate and form functional and mature hepatocytes [77]. In conjunction with the above work, Takebe and colleagues used human iPSCs to generate 3D liver organoids from human iPSCs on Matrigel. The results of these studies demonstrated the successful experimental generation of functional 3D liver organoids [78]. In 2013, Lancaster and colleagues set out to develop a 3D system that could be used to generate 3D cerebral organoids in vitro. This study was conducted using human derived ESCs. These organoids were grown on Matrigel with various neural differentiation media and generated in a spinning bioreactor flask. The obtained results indicated that it is possible to replicate and mimic some elements in the human brain that are involved in neurodevelopment and neurological diseases using a suspended liquid in vitro culture system. The obtained results therefore serve to provide insight into understanding the pathogenesis of neurological disorders [79]. In 2013, Clevers’ Laboratory was the first to report on the generation of pancreatic organoids from mice. The obtained results in this study suggest that pancreas organoids are able to differentiate into endocrine and duct cells post transplantation [80]. In 2015 Huang et al. generated pancreatic organoids from human ESCs. [81]. In both methods the organoids were developed on a Matrigel, and media supplemented with different growth factors [80, 81]. The results of this study were used as a basis for the development of new therapeutic agents for the treatment of pancreatic ductal adenocarcinoma [81]^.^

In 2015, Morizane et al. and Takasato et al. both developed the culture procedure for the generation of kidney organoids from human iPSCs. The cells were grown on ultra-low-attachment plates and Matrigel-coated plates. The obtained results in both studies demonstrated that human iPSCs could differentiate into functional 3D kidney organoids [82, 83]. The use of a spinning bioreactor flask to generate 3D kidney organoids from human PSCs was proven successful in 2021. Przepiorski et al., developed a simplified method to generate organoids. In this instance kidney organoids were generated using human PSCs that were cultured on ultra-low attachment plates and then transferred to a spinner flask. This method proved to be rapid, cost-effective, and efficient in generating large quantities of organoids [84]. In 2018, Turco and colleagues reported the generation of trophoblast organoids that were used to study placenta development, and the investigation of the interactions of trophoblast with the maternal system. In this study human derived tissue was used to generate organoids on a specific culture followed by culturing on Matrigel [85]. In the same year Haider et al. also demonstrated the generation of trophoblasts from tissue samples obtained from multiple patients using first trimester cytotrophoblasts. Organoids were developed on a Matrigel matrix in cell culture plates [86]. In 2019 Wimmer et al. developed the first human blood vessel organoid. Human iPSCs were differentiated into organoids on ultra-low attachment plates to form aggregates and embedded in Matrigel. The results obtained for this study demonstrated that tissue culture generated organoids could recapitulate the function and structure of human blood vessels [59]. Many more organoids have been developed over the years from mice, human iPSCs and ASCs [48, 87]. To date there are various organoids that have been generated from multiple organs mainly; brain [9, 88], kidney [89–91], lung [92, 93], pancreas [81, 94, 95], intestine [73, 96], stomach [97, 98], liver [99], blood vessel [100] and skin [101, 102] (Fig. 2B).

## Organoid Culture Techniques

**Fig. 3:**
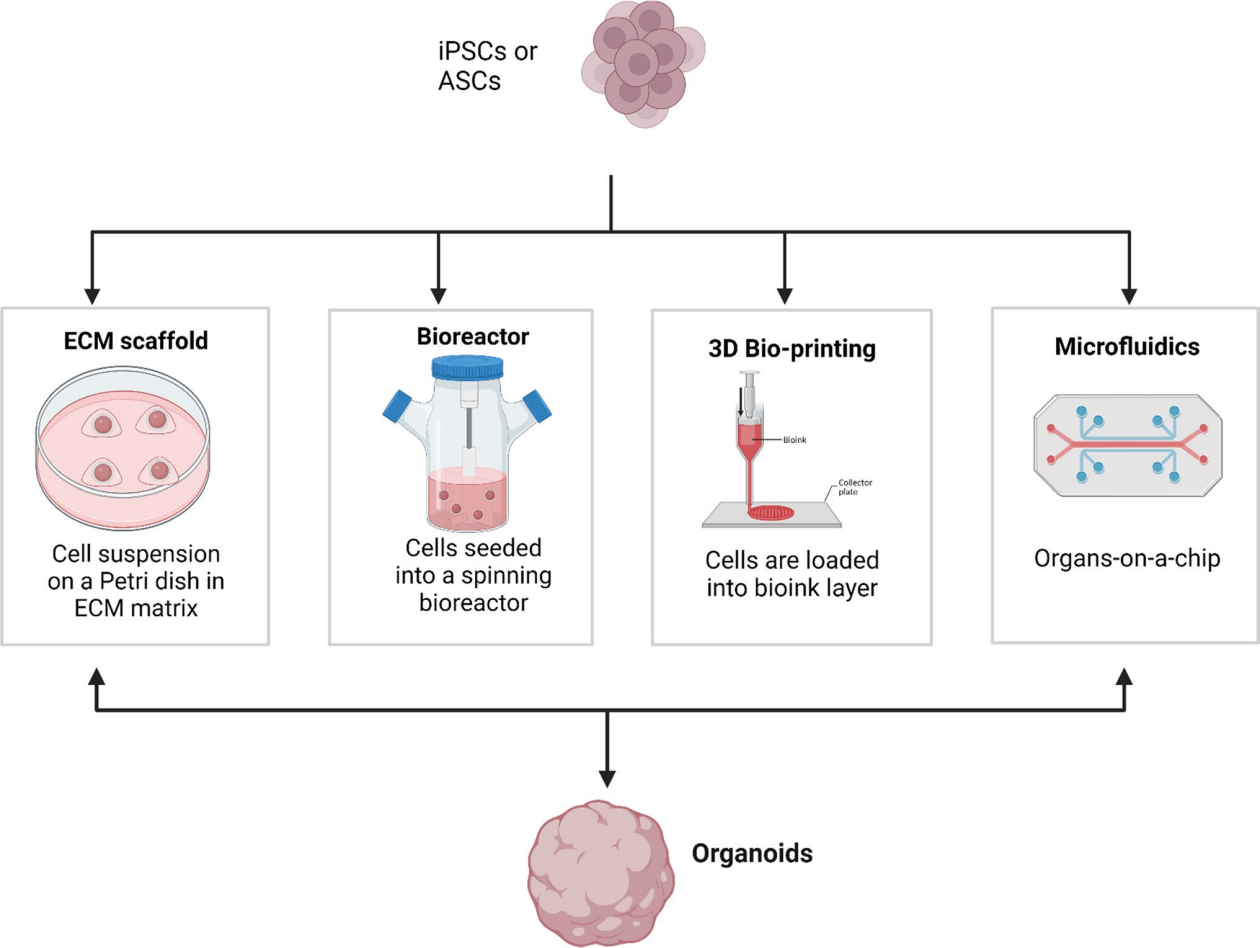
Schematic diagram on methods that have been identified to generate organoids. Organoids have been generated from induced pluripotent stem cells (iPSCs) and adult stem cells (ASCs) using various techniques

Organoids can be generated from cells that are tissue derived or iPSCs. Various methods have been developed for the replicating of the organoid microenvironment that would allow for organoid growth and development. Over the years various organoids have been generated from PSCs and ASCs using various techniques, which include the use of stirred bioreactors [24], extracellular matrix scaffolds [103], 3D bioprinting [22], and using organoid-on-a-chip [22] also see (Fig. 3).

### Extracellular Matrix Scaffold

Using an extracellular matrix scaffold is another method used for generating organoids, in a synthetic or natural environment to induce biological processes such as tissue proliferation, organization and migration [103]. This method was developed by the Hans Clever’s team, in brief ASCs are plated on an ECM Matrigel, and maintained under the selected culture conditions. The use of ECM is coupled with the use of either a hydrogel or Matrigel. This method generates organoids that conform genetically and phenotypically to recognizable organoids structures [63, 104, 105]. The use of Matrigel has been one of the widely accepted ECM techniques, this is mainly because Matrigel provides structural support for the organoid tissue as well as cell differentiation factors [106, 107].

### Culture Using Bioreactors

The principle of this technique is to use cell suspension culture in a stirred medium to develop organoids by agitation or increasing media velocity in place of culturing cells on a solid media in petri dishes [24]. Different bioreactor configurations have been used for generating organoids by growing suspension cell culture based on either rotational (bioreactor) or spinning (spinning flask) and are differentiated by the amount of shear force applied to the cell culture [96]. The spinning flask suspension culture makes use of a stirring bar or rod while rational bioreactors rotate the culture container [24, 107]. Suspension cell bioreactors increase the formation of organoids from embryoid bodies under controlled physical conditions [108, 109]. The use of these bioreactor systems allows for adequate diffusion of nutrients and oxygen to organoid cells because of constant mixing of the liquid cell culture medium, allows simple media exchange [79, 110, 111]. It has been shown that the use of bioreactors also increases organoid culture longevity, differentiation yield and the development of complex [112].

### Three-Dimensional Bio-Printing

Three-dimensional organoids can be generated through bioprinting, in this technique loads stem cells are loaded into bioinks for layer-to-layer deposition to form 3D constructs of organoids [22, 103]. Primarily Hydrogels are primarily used as ink where primary cells are induced with different induction media and then printed on trans wells or perfused microwells to induce the generation of organoids [113]. Bioprinting uses spatial architecture design for PSCs or ASCs, enabling high-precision and high-throughput organoid formation [114]. The use of 3D bioprinting requires the use of growth factors, stem cells and computers to generate 3D structures [114, 115].

### Micro-Fluidics

The micro-fluidics technique is based on the use of culture devices that allow for the fabrication of “organoids-on-a-chip” and allow for nutrient exchange and controls the 3D micro-environment [107]. This technique has been developed to allow for the creation of an environment in which different cell types interact with one another [116]. Using this technique, a more precise model of host–pathogen interactions has been made possible in studying infectious diseases thus providing understanding of the pathophysiology of infectious micro-organisms [117].

## Applications of Organoids in Regenerative Medicine

Stem cells are seen as an important component of regenerative medicine because they provide structural and functional components. Stem cells have been used to generate biological tissue in vitro in regenerative medicine [118]. The field of regenerative medicine aims to restore tissue to its’ normal structural form and function post injury. Regenerative medicine has previously focused on the paradigm of replacement, regeneration and rejuvenation whilst looking at bridging the gap between advances in stem cell therapy and individualized disease management [119]. Replacement focuses mainly on the transplantation of cell-based therapy, regeneration focusing on engraftment of progenitor cells and rejuvenation entails activating endogenous stem cells to promote tissue self-renewal [120]. A breakthrough of regenerative medicine has been the generation of organoids. Organoids are 3D aggregates of stem cells derived from specific organs [121]. The use of organoids has advanced applications in regenerative medicine, mainly in tissue engineering and stem cell therapy, involving organogenesis and transplantation of organoids [10]. Organ transplantation as a treatment of any disease is still constrained by a few limitations which include organ shortage and rejection risk from the affected patients. However, the use of organoids in transplantation therapy is proving to be a promising approach in modelling cancer treatment as a major component in drug discovery and molecular mechanism analysis [122]. With advances in organoid technology and generation, organoids are being demonstrated to play a pivotal role [123, 124] Fig. 2, shows the different organoids that have been engineered and the methods that have been used concurrently.

Organogenesis is the process by which new organs are formed from germ layers. There are three identified germ layers consisting of the ectoderm, mesoderm and endoderm [47, 50]. Organoids are derived from stem cells that are either pluripotent or adult derived. Adult stem cells are derived from specific tissues and the generation of the specific organoids [47, 125]^.^ Pluripotent stem cells require differentiation into the different germ layers as a building block toward organoid genera [126]. Over the years many researchers have advanced and modelled various diseases from organoids and performed transplantations in animals such as mice and rats [18, 27, 46]. In 2018, Daviaud and colleagues performed the transplantation of cerebral organoids derived from human iPSC into mouse cortex. This study was performed to model diseases that affect the central nervous system. The obtained results indicated successful transplantation and engraftment of cerebral organoids [127].

**Fig. 4:**
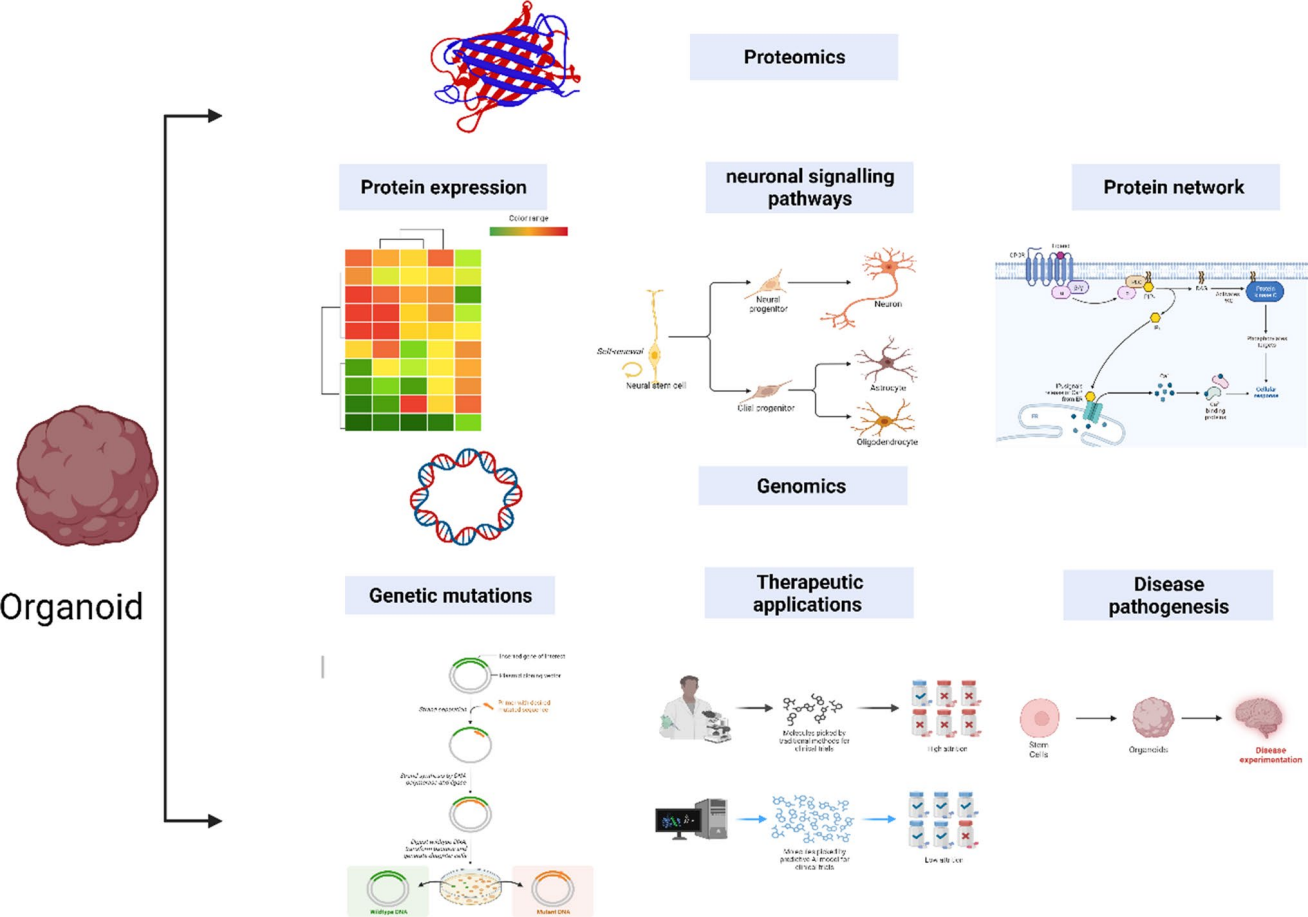
Applications in proteomics and genomics profile of organoids

Proteomics and genomic technologies have significantly impacted regenerative medicine by providing insights into the identification of key biomarkers, signalling molecules, and pathways essential for understanding organoid behaviour in various therapeutic contexts [128, 129]. These technologies have significantly changed the analysis of various mechanism underlying understanding organoids mechanism of proteins [128], genes [53] and signalling pathways [46]. Proteomic techniques enable the comprehensive examination of all the proteins expressed in organoids, providing a comprehensive picture of their functional components Involved in understanding protein changes, interactions and modifications [128]. Conversely, genomic technologies facilitate the interpretation of the genetic blueprints that underlie the growth and behaviour of organoids by identifying mutations genetic alterations as well as DNA analysis [130, 131]. To date advancement to the application of genomic and epigenomic applications in organoids has been reviewed in details by Nam and colleagues [132]. Figure 4 summarizes various applications used in proteomic and genomic technologies used in organoid generation and profiles.

## Clinical and Pre-Clinical Models of Three-Dimensional Organoids

The study of disease aetiology and the discovery of new drug targets have both benefited from the use of 3D organoids as pre-clinical models [133]. The recent development of organoid models has paved the way for cutting-edge alternatives to animal-based research [134]. More than 90% of medicines that enter human clinical trials fail due to safety or efficacy issues, raising the question of whether human benefits outweigh the costs of animal [134, 135]. In 2020, Narsimhan and colleagues performed an experiment to figure out if organoid testing would assist in patients undergoing treatment for peritoneal metastases. In this study colorectal peritoneal metastases organoids were generated using patient derived samples. This study was focused on patients who were receiving various treatments, and the study was performed using various drugs for screening. This research set the path for a phase II clinical trial to assess the effectiveness of this organoid-based platform in providing individualized therapy to patients with colorectal peritoneal metastases [136]. In 2022, Westerling-Bui and colleagues set out to develop a new approach to study pharmacodynamics and overcome the challenges that are being faced with using animal models. They identified that animal models provide uncertain applicability to human conditions when looking at kidney treatment. In this study, GFB-887, a new drug that was in phase II of the clinical trial, was orally dosed into a rat that had previously undergone kidney organoid transplantation. The results from this study showed that pharmacodynamics studies using organoids transplanted in rat host could serve to provide insight into the assessment of pre-clinical efficacy, as pre-clinical efficacy was reached in this study [137]. Based on the work performed by Narasimhan et al. and Westerling-Bui et al. novel drugs are currently being tested in clinics (Table 2).

**Table 2:** Organoids clinical trial reportss

Organoid	Source	Disease Modelled	Experimental Aim	in vivo or in vitro	Observation	Limitations	Reference
Prostate	Patient derived	Prostate cancer	Understanding the pathophysiology of prostate cancer and the effectiveness of treatment using 3D organoids	In vitro	Patient derived organoids can be used for drug studies in vitro and as xenografts in vivo studies		[64]
Liver	Human iPSCs	Liver failure	Generating functional and vascularized liver	In vitro	Functional vascularized organoids were generated, and organ-bud transplantation can be used as an alternative approach to generate vascularized organoids	Method development and standardization required prior to patient treatment Animal models are still required to determine vascularization or organoids	[78]
Alveolar	Human PSCs	Idiopathic pulmonary Fibrosis (IPF)	To determine whether hPSC-derived fibroblast-dependent alveolar organoids could be useful for drug screening and therapeutic target identification for pulmonary fibrosis, focusing on alveolar epithelial cells	In vitro	Fibroblast-dependent alveolar organoids may be useful for screening therapeutic compounds to treat IPF and are able to mimic the interactions between human alveolar epithelial cells and fibroblasts in pulmonary fibrosis in vitro	No clear indication if alveolar epithelial cell-specific damage could activate fibroblasts in fibroblast-dependent alveolar organoids	[138]
Thyroid	Patient derived tissue	Papillary thyroid cancer	To demonstrate how papillary thyroid cancer organoids can be used as a promising novel preclinical model for representing individual patients	In vitro	The use of patient derived papillary thyroid cancer organoids allowed for efficacy testing of anticancer drugs in individual patients	The study focused on patients who underwent surgery between 2019 and 2020 and showed no disease progression	[139]
Liver	Human iPSC and ESC		Generate hepatic organoids from human iPSCs with high drug metabolic ability	In vitro	Generated human hepatic organoids model provides tools for drug testing and disease modeling		[140]
Rectal	Patient derived cells through biopsy		A living organoid biobank was generated from patients with locally advanced rectal cancer and were treated with neoadjuvant chemoradiation	In vitro	Rectal cancer organoids accurately replicate the pathophysiology and genetic changes of corresponding tumors	-	[141]
Midbrain	iPSCs	Parkinson’s disease	To investigate the patho-mechanism of Parkinsons disease in patients with LRRK2-G2019S mutation using chemically derived midbrain floor plate neural progenitor cells	In vitro	Patient derived 3D human midbrain-specific organoids and midbrain floor plate neural progenitor cells generated are potent tools in in vitro disease modelling for personalized medicine techniques	The use of 3D models allows for studying neurodegenerative diseases require replicating the neuron-neuronal interaction and to date no brain organoid generated show Parkinsons disease symptom	[142]

## Limitations of Organoid Application

The generation of organoids has significant promise in personalized treatment, tissue engineering, drug discovery and disease modelling [143]. However, there are still some limitations and restrictions in using organoids. In this section we will be looking at the current limitations and the future directions that could be considered.

### Technique and Protocol

Organoids are generated using different techniques, as such there is no specific protocol on the generation of organoids that has been developed. The morphological development of organoids may be restricted by naturally produced ECMs like Matrigel due to batch-to-batch variability and the presence of animal-derived products [143, 144].

### Lack of Vascularization

Organoids are generated from stem cells of specific tissues however, despite their specific derivation organoids, lack vascularization, neural and blood flow networks [143], which allows for adequate oxygen and nutrient exchange during organogenesis. Scientists have developed ways to generate vascularized organoids both in vitro vascularization and in vivo vascularization [145, 146]. In vitro vascularization is a developed technique performed by adding vascular cells or tissue engineering by use of bio-printing [143, 146]. In vivo vascularization of organoids has been achieved by organoid transplantation into animal model [146].

### Maturation and Functionality

Often generated organoids are small and range from 100 µm to 300 µm, making it difficult to work with during in vivo applications [143]. It has been found that the use of bioreactors in suspension media containing various growth supplements allows for large quantities of organoids to be generated at a size of up to 1 mm [79, 147]. Bioreactors have been seen to increase the number of organoids, photoreceptor cell yield, increased proliferation and decrease in apoptosis [148].

### Ethical Issues

Organoids suggest significant promise for a wide range of biomedical and biotechnological applications. Regardless of its scientific potential, organoid technology presents difficult ethical challenges that could prevent any future benefits for patients and society [149]. Based on the generation of various organoids, different ethical issues have been reported [150]. One of the concerns regarding the generation of organoids is that they grow from ESCs [149]. Previously, animal models were used as a proxy for human embryonic development and organ function research, however, the generation of organoids using ESCs has major ethical concerns [149, 151, 152]. The use of iPSCs has the potential to serve as an alternative as intestinal organoids were previously developed from iPSCs [73]. The generation of cerebral organoids has also developed many ethical issues. It is unknown whether brain organoids, which are neuronal entities of human origin, can acquire human traits, cognitive functions, or sentience [153].

## Conclusion and Future Perspectives

In conclusion, the use of 3D organoids in stem cell therapy, offers significant promise for developing the area of regenerative medicine. Organoids offer an effective foundation for researching organ development, disease modelling, and identifying the effectiveness of stem cell-based therapies. The use of 3D organoids allows researchers to study stem cell regeneration characteristics and interactions within the appropriate physiological environment [53]. This provides for better understanding of tissue regeneration mechanisms and the development of novel techniques. The use of organoids has the potential to overcome numerous research challenges in modelling diseases and bridging the gap between pre-clinical studies and clinical application.

The future of regenerative medicine will be dependent on improving 3D organoid culture methods to resemble the complexity and functionality of actual organs and tissues more closely. The identification of novel techniques for inducing the development of organoids with capacities for vascularization and immune responses will also provide insight into the physiology underlying these responses and their regenerative potential. There are still a few outstanding issues that need to be resolved before stem cell-based organoids can be used in a clinical setting. This includes increasing the production of organoids to clinically relevant quantities [7]. This would include developing large bioreactors that would allow for large organoid generation, and developing automated culture systems that would limit human error and make the process less time consuming [148]. Developing a standardized and reproducible protocol for the generation of organoids is a pre-requisite for the clinical application of organoids [48]. The absence of a standardized approach that is reproducible increases challenges that affect the functionality and quality of generated organoids. Although organoids mimic the actual organ or tissue, the functionality of the organoids remains a challenge as there is no set way to determine whether the generated organoids can function as required [53, 154]. Despite the present challenges identified with organoids there is immense therapeutic potential for numerous disease treatment.

## Data Availability

Not applicable.
